# The prevalence of stillbirths: a systematic review

**DOI:** 10.1186/1742-4755-3-1

**Published:** 2006-01-10

**Authors:** Lale Say, Allan Donner, A Metin Gülmezoglu, Monica Taljaard, Gilda Piaggio

**Affiliations:** 1 UNDP/UNFPA/WHO/World Bank Special Programme of Research, Development and Research Training in Human Reproduction, Department of Reproductive Health and Research, World Health Organization, Geneva, Switzerland; 2 Department of Epidemiology and Biostatistics, Faculty of Medicine and Dentistry, University of Western Ontario, London, Canada; 3 Robarts Clinical Trials, Robarts Research Institute, London, Canada

## Abstract

**Background:**

Stillbirth rate is an important indicator of access to and quality of antenatal and delivery care. Obtaining overall estimates across various regions of the world is not straightforward due to variation in definitions, data collection methods and reporting.

**Methods:**

We conducted a systematic review of a range of pregnancy-related conditions including stillbirths and performed meta-analysis of the subset of studies reporting stillbirth rates. We examined variation across rates and used meta-regression techniques to explain observed variation.

**Results:**

We identified 389 articles on stillbirth prevalence among the 2580 included in the systematic review. We included 70 providing 80 data sets from 50 countries in the meta-analysis. Pooled prevalence rates show variation across various subgroup categories. Rates per 100 births are higher in studies conducted in less developed country settings as compared to more developed (1.17 versus 0.50), of inadequate quality as compared to adequate (1.12 versus 0.66), using sub-national sample as compared to national (1.38 versus 0.68), reporting all stillbirths as compared to late stillbirths (0.95 versus 0.63), published in non-English as compared to English (0.91 versus 0.59) and as journal articles as compared to non-journal (1.37 versus 0.67). The results of the meta-regression show the significance of two predictor variables – development status of the setting and study quality – on stillbirth prevalence.

**Conclusion:**

Stillbirth prevalence at the community level is typically less than 1% in more developed parts of the world and could exceed 3% in less developed regions. Regular reviews of stillbirth rates in appropriately designed and reported studies are useful in monitoring the adequacy of care. Systematic reviews of prevalence studies are helpful in explaining sources of variation across rates. Exploring these methodological issues will lead to improved standards for assessing the burden of reproductive ill-health.

## Background

The use of perinatal deaths to assess pregnancy outcomes has been a practical approach particularly in settings and circumstances where it is not always easy to distinguish between stillborn and liveborn infants who die shortly after birth. However, due to difficulties in measurement and etiological differences between the two components of perinatal deaths – stillbirths and early neonatal deaths – its value is limited [1,2]. Separate measure of stillbirths as an indicator of access to and quality of antenatal and delivery care, therefore, is becoming increasingly important. The higher stillbirth rates shown among lower socio-economic groups of populations in both developing and more developed parts of the world [3-5] extend the use of stillbirth rate as a development indicator as well.

Obtaining reliable estimates of stillbirth rates and making cross-country comparisons has been problematic for several reasons. Routine vital registration information is suggested to be an underestimate of the true picture particularly in developing countries [6,7]. Community surveys rely on self-reports which may not always be valid [8]. Relying only on facility-based data may be misleading since considerable number of deliveries occur at home in many developing country settings. A variety of definitions and cut-off levels for registration involving different gestational ages ranging from 20 to 28 weeks or birth weights ranging from 350 to 1000 g further complicates interpretation of rates [1,9-15].

In addition to routinely collected data, medical literature includes a range of studies reporting on the prevalence of stillbirths. The results of these studies show variation across and within countries. For example, per 1000 live births, it has been reported as 61 in Zimbabwe, 18 in Turkey and ranging from 3.2 to 7.1 among different ethnic groups in Canada all using the definition involving birth weight of more than 500 g [4,16,17].

Although there is a wealth of information through routine registration systems and a variety of ad hoc studies, due to the complexities described above, reliable estimates of stillbirth rates do not exist for many settings. Rates vary across and within settings, and summarizing outcomes is not always straightforward. Meta-analytical methods are increasingly being used in comparing and summarizing outcomes for important public health outcomes. They offer valuable tools, particularly for research carried out across different settings, by providing an opportunity to investigate potential sources of variation [18].

We conducted a systematic review and performed meta-analysis of available information from both routine data and other published studies to explore the feasibility of obtaining an overall estimate of the stillbirth rates across various regions of the world and to investigate possible sources of heterogeneity across these rates.

## Methods

This study is the analysis of the stillbirth component of the systematic review of maternal mortality and morbidity undertaken by the UNDP/UNFPA/WHO/World Bank Special Programme of Research, Development and Research Training in Human Reproduction (HRP), Department of Reproductive Health and Research at the WHO. The objective of the systematic review was to obtain prevalence/incidence data on maternal mortality and a range of conditions including stillbirths. The detailed methodology which followed a pre-defined protocol has been described elsewhere [19].

### Identification of the articles

The search for articles involved bibliographic databases (Medline, EMBASE, SocioFile, CAB Abstracts, Econlit, Cinahl, LILACS, Popline, BIOSIS, PAIS), WHO regional databases (African Index Medicus, Index Medicus for the Eastern Mediterranean Region), internet, reference lists, contacting experts in the field, and hand-searching of relevant documentation in the WHO Library. We developed specific search strategies for electronic databases according to their structured thesaurus terms or using appropriate keywords in collaboration with two librarians from the WHO and Cochrane Pregnancy and Childbirth Group. Detailed strategies for electronic databases have been previously reported and are available from the authors [19]. The search was limited to articles dated from 1997 to 2002. The decision for this was arbitrary. There were no language restrictions.

### Assessment for inclusion

Two reviewers evaluated titles and abstracts of the identified citations for potential inclusion in the review. Prior to this initial evaluation, we assessed inter-observer agreement using the kappa statistics (0.60 95% CI 0.52 to 0.69) which showed moderate to substantial agreement [20]. We discussed and resolved points of disagreement. In case of doubt, we obtained full text articles of citations. We assessed full-texts of the articles deemed to be potentially relevant at the initial stage. Studies in all languages were eligible for inclusion if they reported data relevant to outcomes of interest, specified dates for data collection period, included data from 1990 onwards, and had sample sizes of greater than 200.

### Data extraction and quality assessment

We developed and used a data extraction instrument including 48 items distributed in five modules three of which were relevant to this analysis. Modules were designed to collect information on (i) study level characteristics (sampling design, population, setting, completeness of data/response rate, reference period), (ii) outcome measures, and (iii) definitions and identification procedures for outcomes. We defined four key criteria for the quality assessment of the articles. These were: sampling schemes conducted as either random or consecutive, adequate description of population characteristics, definition of both the numerator and the denominator of the reported rate, and response rate/completeness of information in the data sets exceeding 75%. We considered the overall quality as adequate if a study fulfilled at least three of the four criteria. We did not exclude studies on the basis of inadequate quality, but accounted for this in the statistical analysis.

### Selection of studies

Prior to the analysis, we developed a protocol that defined inclusion criteria and specified the approach to the analysis. Cross-sectional studies reporting stillbirth rates with representative sampling schemes were eligible for inclusion. For studies reporting information relevant to the same population for more than one year, we included data only from the most recent year. In order to prevent a woman's appearance more than once in a data set, and because the durations of studies extending beyond 12 months were highly variable, we limited analysis to studies with reference periods of 12 months. For studies where no definition for stillbirth was reported we assumed the conventional definition of more than 28 weeks of gestation [1]. If a study reported results separately for different definitions, we used data referring to the conventional definition.

### Statistical procedures

We calculated the pooled prevalence estimates for various subgroup categories weighted by the sample size of individual studies. A meta-regression was conducted to identify significant sources of heterogeneity [21].

The independent study-level variables included in the meta-regression were as follows: development status of the country where the study was conducted (developed versus less/least developed according to the United Nations classification system [22], definition of numerator of stillbirth rate (late stillbirths – more than 28 weeks gestation or more than 1000 g birth weight versus all stillbirths – other categories involving earlier gestational ages starting from more than 20 weeks or birth weight more than 500 g), definition of denominator of stillbirth rate (live births versus pregnancies/deliveries), overall quality of the study (adequate versus inadequate), scope of study (national versus sub-national), source (journal versus non-journal) and language of the article (English versus non-English).

For the purposes of statistical inference, the prevalence rates were transformed using the empirical logistic transformation [23] given by



where *a*_*i *_is the numerator of the prevalence rate, and *n*_*i *_is the denominator. This transformation is used to help normalize the distribution of the dependent variable in preparation for the subsequent regression analyzes. The estimated inverse variance was used as weight in these analyzes, where the variance is given as:



For studies using a multistage design, this variance was estimated as :



where *deff *is the estimated design effect [24] for neonatal mortality.

The SAS Procedure REG, was used to conduct the weighted least squares regression [25]. The option BACKWARD was specified to allow selection of the subset of independent variables that best predict the dependent variable. This procedure first fits a model with all the candidate variables included, followed by the deletion of variables in a stepwise fashion. The level of significance for stepwise removal from the model was set at 0.10.

## Results

**Figure 1:**
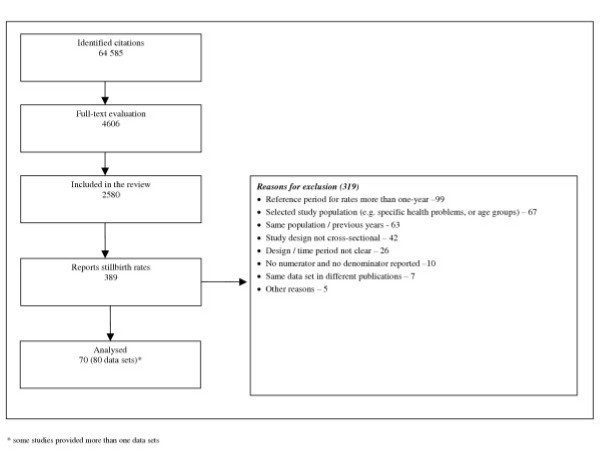
Flow diagram of identification of studies.

**Figure 2:**
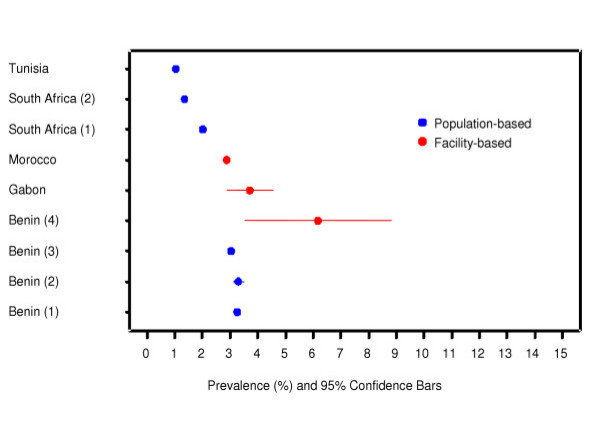
Stillbirth prevalence: Africa.

**Figure 3:**
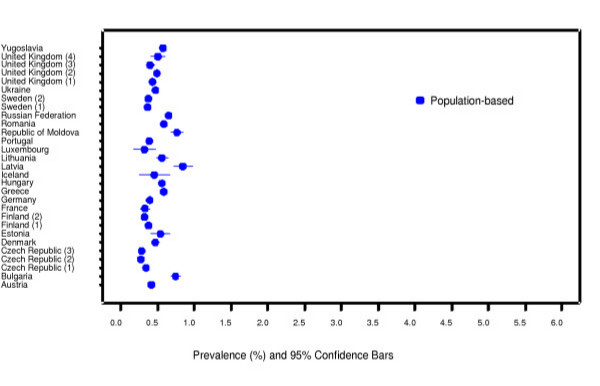
Stillbirth prevalence: Europe.

We identified a total of 64 585 articles and included 2580 in the systematic review of which 389 reported stillbirth rates. We excluded 319 according to the pre-specified criteria for this analysis (figure 1). A total of 70 studies providing 80 data sets from 50 countries were analysed. Among these 80 data sets, 63 were population [26-78] and 17 were facility-based [79-95]. Graphical representations for country-specific prevalence rates for two regions – Africa and Europe are presented in figures 2 and 3, respectively.

Descriptive characteristics for the population-based and the combined sets of studies are presented in table 1. About half the population-based studies took place in less developed country settings and the majority included national (78%) rather than sub-national data. Quality was adequate in 65% of the population-based studies. Population-based data sources were mostly non-journal reports (84%) and stillbirths were defined as late stillbirths in 75%. All population-based studies and 16 of the facility-based studies reported their use of consecutive sampling to select the sample of subjects. A facility-based study was more likely to be in a journal article format, of inadequate quality and from a developing country setting.

**Table 1:** Description of data sets

	Population-based data sets (N = 63)	Combined (Population and facility-based) data sets (N = 80)
Characteristic	N (%)	

Development status^1^		
Less/least developed	31 (49.2)	47 (58.8)
Developed	32 (50.8)	33 (41.3)
Year study began		
Before 1999	23 (36.5)	37 (46.3)
After 1999	40 (63.5)	43 (53.8)
Source		
Non-journal article	53 (84.1)	55 (68.8)
Journal article	10 (15.9)	25 (31.3)
Language		
Non-English^2^	36 (57.1)	46 (57.5)
English	27 (42.9)	34 (42.5)
Sampling design		
Consecutive	63 (100.0)	79 (98.8)
Unknown		1 (1.3)
Definition of numerator		
Late stillbirth	47 (74.6)	61 (76.3)
All stillbirth	16 (25.4)	19 (23.8)
Denominator definition		
Other^3^	9 (14.3)	18 (22.5)
Live birth	54 (85.7)	62 (77.5)
Quality		
Inadequate	22 (34.9)	33 (41.3)
Adequate	41 (65.1)	47 (58.8)
Type		
Facility-based	NA^4^	17 (21.3)
Population-based	63 (100.0)	63 (78.8)
Scope		
Sub-national	14 (22.2)	NA
National	49 (77.8)	NA

^1^United Nations classification is used

^2^Includes reports in original language accompanied with English or French

^3^Delivery or pregnancy

^4^Not applicable

Pooled prevalence rates (per 100) for various subgroups of the population-based studies are shown in table 2, where the substantial heterogeneity among sub-regions stands out clearly. Also notable is the large difference in rates between developed and less/least developed countries (0.50 versus 1.17) and between studies having adequate versus inadequate quality (0.66 versus 1.12).

**Table 2:** Pooled stillbirth rates in subgroups of population-based data sets

	Number of data sets	Median study size	(Pooled) rate/100
Overall	63	76 982	0.84
Development Status^5^			
Developed	32	75 974	0.50
Less/Least Developed	31	76 982	1.17
Quality			
Inadequate	22	75 331	1.12
Adequate	41	78 268	0.66
Scope			
Sub-national	14	37 618	1.38
National	49	90 446	0.68
Source			
Non-journal article	53	76 982	0.67
Journal Article	10	77 829	1.37
Language			
Non-English^6^	36	98 971	0.91
English	27	41 451	0.59
Numerator Definition			
Late stillbirth	47	89 928	0.63
All stillbirth	16	59 166	0.95
Denominator Definition			
Other^7^	9	70 687	0.83
Live Birth	54	83 436	0.85
Subregion^8^			
Northern Africa	1	158 486	1.06
Southern Africa	2	94 591	1.79
Western Africa	3	148 267	3.19
Eastern Asia	2	610 588	0.44
South-Central Asia	7	104 762	0.56
South-Eastern Asia	4	42 394	0.36
Western Asia	3	13 437	0.73
Eastern Europe	9	90 715	0.59
Northern Europe	13	56 189	0.46
Southern Europe	3	119 368	0.52
Western Europe	4	53 854	0.40
Caribbean	2	79 864	0.91
Central America	2	41 724	0.82
South America	6	231 712	1.34
Australia/New Zealand	2	37 594	0.64

^5^United Nations classification is used

^6 ^Includes reports in original language accompanied with English or French

^7 ^Delivery or pregnancy

^8 ^United Nations classification is used

Other emerging patterns seen from table 2 are that the higher pooled rates are found in studies using sub-national sample as compared to a national sample (1.38 versus 0.68), reporting all stillbirths as compared to late stillbirths (0.95 versus 0.63), published in non-English as compared to English (0.91 versus 0.59) and as journal articles as compared to non-journal articles (1.37 versus 0.67).

The results of the meta-regression are presented in table 3. The analysis of population-based studies show that development status and study quality are the only variables showing statistical significance at the 5% level in the final step down model. Thus, more developed regions have lower prevalence rates on the average than less developed regions of the world (95% CI for adjusted relative odds (0.33, 0.57)), and studies of adequate quality have lower prevalence rates on the average than studies of inadequate quality (95% CI for adjusted relative odds (0.56, 0.94)). The *R*^2^-value, which is an overall measure of how well the independent variables (development status and study quality) together, predict the dependent variable (stillbirth rates), is 52.4%.

**Table 3:** Meta-regression results

***Population-based data sets (N = 63)***		
**Full Model**		

**Predictor**	**Relative odds (95% CI)**	**p-value**

Development status (dev vs. less/least dev)	0.46 (0.34, 0.62)	<0.001
Source (journal vs. non-journal)	1.01 (0.65, 1.58)	0.966
Language (English vs. non-English)	0.76 (0.51, 1.13)	0.169
Numerator definition (all vs. late)	1.08 (0.75, 1.54)	0.686
Denominator definition (live birth vs. other)	0.71 (0.37, 1.34)	0.281
Quality (adequate vs. inadequate)	0.71 (0.49, 1.03)	0.067
Scope (national vs. sub-national)	0.97 (0.63, 1.47)	0.868
Model R^2 ^= 0.527		

**Final Model**		

Development status (dev vs. less/least dev)	0.43 (0.33 ; 0.57)	<0.001
Quality (adequate vs. inadequate)	0.73 (0.56 ; 0.94)	0.015
Model R^2 ^= 0.524		

***Combined data sets (Population and facility-based) (N = 80)***		

**Full Model **		

Development status (dev vs. less/least dev)	0.45 (0.34, 0.58)	<0.001
Source (journal vs. non-journal)	1.00 (0.75, 1.31)	0.973
Language (English vs. non-English)	0.76 (0.54, 1.05)	0.092
Numerator definition (all vs. late)	1.07 (0.77, 1.47)	0.690
Denominator definition (live birth vs. other)	0.71 (0.43, 1.19)	0.189
Quality (adequate vs. inadequate)	0.70 (0.50, 0.97)	0.033
Population vs. facility-based	0.50 (0.38, 0.78)	0.001
Model R^2 ^= 0.638		

**Final Model**		

Development status (dev vs. less/least dev)	0.43 (0.33, 0.55)	<0.001
Quality (adequate vs. inadequate)	0.73 (0.58, 0.92)	0.007
Population vs. facility-based	0.54 (0.39, 0.73)	<0.001
Model R^2 ^= 0.621		

The analysis of the combined study file supports the findings of the population-based meta-regression analysis, showing that three of the predictor variables are significant at the 5% level, namely development status, study quality, and whether or not a study is population-based. Hence, population-based studies have lower prevalence rates on the average than facility-based studies (95% CI for adjusted relative odds (0.39, 0.73). This is consistent with the relatively large difference in unadjusted overall prevalence rates for the population-based and facility-based studies, given by 0.84 and 2.50 respectively.

Tests for interaction effects performed as a secondary analysis revealed a significant interaction between development status and study quality both in the in the population-based file (p = 0.038) and in the combined data file (p = 0.018). This interaction arises because the effect of *study quality *on stillbirth prevalence rates in more developed regions is different from the effect in less developed regions of the world. In particular, studies of adequate and inadequate quality tend to exhibit smaller differences in prevalence rates in more developed regions than in less developed regions of the world.

The results obtained from residual analysis of the final models revealed no evidence of departure from standard underlying assumptions, namely that the residuals are independent, have a common variance with mean 0 and follow a normal distribution.

## Discussion

Our results suggest that stillbirth prevalence at the community level is in general less than 1% in more developed parts of the world and could exceed 3% in less developed regions, but we were not able to provide overall estimates of stillbirth prevalence for different regions of the world due to significant heterogeneity across sub-regions. Facility based studies show higher rates, which could be due to referral bias.

Meta-regression analysis explained a considerable proportion (52%) of the observed heterogeneity in these data. Not surprisingly, development status of the setting in which the study was conducted was shown to be a strong predictor of stillbirth prevalence. Perhaps less expected was that the quality of a study is another significant predictor, independent of development status, with prevalence rates being lower in studies of higher quality. All other study-level variables we tested for possible influence on stillbirth rates did not show a significant relationship. The remaining variation could be due to other unmeasured variables that could not be investigated in this analysis. For example, with the information available to us, we could not investigate the influence of characteristics such as age and parity, both of which are important predictors of stillbirth [96-98].

Meta-analytical methods including meta-regression has increasingly been used in summarizing outcomes and explaining between-study variability in investigations of treatment effects or associations [99-101], but its use in prevalence studies is relatively infrequent, with existing literature largely limited to the area of mental health [102,103].

The meta-regression techniques were helpful in explaining a significant portion of the observed variation in stillbirth rates. We believe it is timely to use this approach more widely in the estimation of maternal and perinatal health indicators associated with internationally set goals and targets. The need for global estimates of such indicators is greater than ever in the context of international development goals including the Millennium Development Goals (MDGs) [104]. More empirical evidence should improve the selection, implementation and interpretation of indicators used to monitor the progress towards achievement of the MDGs as well as addressing the increased demand for reliable estimates.

The empirical evidence we provide regarding the significant influence of the development status of the study setting on stillbirth prevalence has implications for policy and programmatic actions. The significantly higher rates in less developed country settings and the highest rate observed in Western Africa could largely be due to inadequacies in accessing appropriate maternal health care during both antenatal period and delivery. The reported skilled attendance at birth in this region is also very low, corroborating these findings [105].

The independent effect of the quality of primary studies on the rates deserves attention as well. For effect-size studies the perceived quality of a published article is known to be related to its likelihood of being included in a meta-analysis [106], although the extent to which this is also true for prevalence studies is less well established. It has also been demonstrated that reporting of observational studies including cross-sectional designs are not in accordance with the desirable standards [107]. Our findings contribute to this literature by demonstrating the influence of quality on the outcome of a prevalence study. More carefully conducted and reported studies are needed if researchers want their findings to be useful for the scientific community as well as to have an influence on policy decisions.

Our study has several limitations. First, our analysis focuses on a subgroup of studies selected from a larger systematic review. The search strategy for the larger review, however comprehensive, did not specifically target stillbirths, and therefore, some relevant studies may have been missed. The trade-off in deciding to limit our investigation to prevalence studies having one-year duration reduced the number of studies included in the analysis. We took this decision because the durations of the remaining studies varied widely and studies of longer duration may have counted multiple pregnancies in the same woman. Since stillbirth may be a recurrent event [108-110], we aimed to avoid including repeating stillbirths in the analysis. Finally, as discussed above, we demonstrated that two important variables influence the stillbirth rates, but the influence of other factors, particularly those measured at the individual level, remains to be investigated.

The results of this systematic review show significant variation in stillbirth rates in different parts of the world and that, even in the settings with the highest standards of maternal and perinatal care, around five out of 1000 newborns will be stillborns.

### Implications for policy and practice

While these findings do not have direct implications for clinical practice, they highlight the relative frequency of stillbirth as an indicator of the quality of service delivery. Even in developed countries the fact that stillbirths constitute close to 1% of all births should alert policy-makers to initiate audit procedures to identify avoidable cases and take action.

### Implications for research

We urge epidemiology community to address the methodological standards as well as reporting of prevalence studies. The application of meta-analytical techniques including meta-regression in summarizing prevalence rates needs further research. The standards for data collection and reporting should be addressed through international consensus.

## Contribution of authors

LS and AMG had the idea, designed and conducted the systematic review. AD, LS, AMG and GP planned the analysis. AD and MT carried out the analysis. LS wrote and AMG, AD, MT, GP contributed to the manuscript.
